# Small Engine, Big Power: MicroRNAs as Regulators of Cardiac Diseases and Regeneration

**DOI:** 10.3390/ijms150915891

**Published:** 2014-09-09

**Authors:** Darukeshwara Joladarashi, Rajarajan Amirthalingam Thandavarayan, Sahana Suresh Babu, Prasanna Krishnamurthy

**Affiliations:** Department of Cardiovascular Sciences, Centre for Cardiovascular Regeneration, Houston Methodist Research Institute, Houston, TX 77030, USA; E-Mails: djoladarashi@houstonmethodist.org (D.J.); ramirthalingamthandavarayan@houstonmethodist.org (R.A.T.); ssureshbabu@houstonmethodist.org (S.S.B.)

**Keywords:** miRNA, cardiac development, cardiovascular diseases, hypertrophy, fibrosis, arrhythmia, cardiac regeneration, stem cells

## Abstract

Cardiac diseases are the predominant cause of human mortality in the United States and around the world. MicroRNAs (miRNAs) are small non-coding RNAs that have been shown to modulate a wide range of biological functions under various pathophysiological conditions. miRNAs alter target expression by post-transcriptional regulation of gene expression. Numerous studies have implicated specific miRNAs in cardiovascular development, pathology, regeneration and repair. These observations suggest that miRNAs are potential therapeutic targets to prevent or treat cardiovascular diseases. This review focuses on the emerging role of miRNAs in cardiac development, pathogenesis of cardiovascular diseases, cardiac regeneration and stem cell-mediated cardiac repair. We also discuss the novel diagnostic and therapeutic potential of these miRNAs and their targets in patients with cardiac diseases.

## 1. Introduction

Heart disease is the number one cause of death for both men and women in the United States [1]. By 2030, heart disease will be the leading cause of death throughout the world [2,3]. Sixteen out of eighty million U.S. adults affected with cardiovascular diseases carry the diagnosis of coronary artery disease. Ischemic injury due to coronary artery disease results in permanent loss of cardiac tissue leading to adverse cardiac remodeling process and diminished contractility contributing to heart failure. At the cellular level, heart failure is associated with a decrease in cardiomyocyte and endothelial cell viability, hypertrophy of existing myocytes, inflammation, mitochondrial dysfunction (altered energetics and reactive oxygen species production), cardiac fibrosis, arrhythmia and vascular defects. At the molecular level, heart development and diseases are controlled by various gene regulatory networks including transcription factors, co-activators and repressors, their corresponding enhancer and promoter elements and chromatin-modifying enzymes.

MicroRNAs (miRNAs) constitute a growing class of non-coding small RNAs, 20–25 nucleotides in length that act as molecular switches of gene expression and are thought to regulate complex cardiac signaling and transcriptional circuits during cardiac development and disease [4,5,6]. miRNAs have been shown to exhibit developmental-stage-specific or tissue-specific expression, suggesting that they might play important roles in many biological processes [7]. Indeed, miRNAs are known to regulate different cellular processes such as proliferation, differentiation, cell metabolism, apoptosis and angiogenesis [8,9,10,11,12,13,14,15,16]. Mechanistically, miRNAs regulate gene expression on the post-transcriptional level by inhibiting the translation of protein from mRNA or by promoting the degradation of mRNA. In the present review, we provide an overview of recent studies highlighting the important role of miRNAs in cardiovascular development and diseases, as well as their potential use for diagnosis, prevention, and treatment of cardiovascular diseases.

## 2. Biogenesis of miRNA and Its Target Sites

The first identified miRNA, lin-4 was discovered in 1993 and was shown to control developmental timing in *Caenorhabditis elegans* by suppressing lin-14 protein expression [17,18]. In humans, more than 60% of all protein-coding genes appear to be conserved miRNA targets [19,20]. Most mammalian primary-miRNAs (pri-miRNA) are transcribed by RNA polymerase II as long precursor molecules containing stem-loop structures (approximately 10 kb in length) [21,22]. Following transcription of pri-miRNA in the nucleus, microprocessor complex containing Drosha (RNase III endonuclease) and RNA-binding protein, DGCR8 (DiGeorge syndrome critical region 8) mediate the processing of the pri-miRNA into precursor-miRNAs (pre-miRNA, ~70 nucleotide hairpin-structures) [23]. The pre-miRNA is exported from nucleus to the cytoplasm (by exportin-5 in a Ran-GTP dependent manner), where it is cleaved by another RNase III endonuclease, Dicer, into mature miRNA (~22–25 nucleotide RNA duplex). This RNA is subsequently unwound by a helicase activity, binds to an Argonaute protein and gets incorporated as single-stranded RNA into the RNA-induced silencing complex (RISC) [24], which directs the miRNA to complementary sites within the 3' UTRs (untranslated region) of target mRNAs leading to translational repression or degradation of the target mRNA [23,25]. Interestingly, miRNAs can act as translational activators as well [26]. Based on computational algorithms, around 60% of human transcripts contain potential miRNA-binding sites within their 3' UTRs [20]. A “seed sequence” in the 5' end of the mature miRNA pairs to nucleotides 2 through 8 at the 3' UTR of target mRNAs [23]. However, miRNAs can interact with 5' UTRs, protein-coding sequences and introns [27]. Furthermore, miRNAs can also localize to the nucleus, where they may regulate transcription/splicing of transcripts, or serve as signaling molecules between two cells through exosome transfer [28]. Although a single miRNA can target many genes, it is possible that multiple miRNAs can regulate a single gene [25,29,30]. These studies suggest that miRNA transcription to maturation involves several coordinated steps and that deregulation in miRNA biogenesis and function might contribute to the development of cardiovascular diseases [31,32].

## 3. Role of miRNAs in Cardiac Development and Diseases

### 3.1. miRNAs in Cardiac Development

During embryogenesis, the heart is the first organ to form and disruption in its development and function results in a variety of congenital disorders [32,33]. The developing heart undergoes a series of transformations resulting in the formation of a multi-chambered heart. Cardiomyocytes play an important role in the growth of the heart prior to birth. The post-natal heart growth is attained mainly through cellular hypertrophy [34,35]. Heart development involves precise and complex interactions and regulation of signaling molecules among diverse cell types from several lineages including: cardiomyocytes, endocardial, epicardial and vascular cells, fibroblasts and cells of the conduction system [36,37,38,39,40]. The role of miRNAs as a critical regulator of biological processes in embryonic, postnatal, and adult hearts is beginning to evolve.

Cardiac cells contain specific types of miRNA and it was found that only a particular miRNA participates in the specification of cell identity. The expression of miRNA-1 and miRNA-133a is cardiac and skeletal-muscle specific. Zhao *et al.* (2007) [31] showed that miRNA biogenesis in the mouse heart is essential for cardiogenesis and that targeted deletion of muscle-specific miRNA, miRNA-1-2, revealed numerous functions in the heart, including regulation of cardiac growth and differentiation, electrical conduction, and cell-cycle control through modulation of transcription factors like Irx4, Hrt2, Hand1 and Gata6. Furthermore, *miRNA-1* and *miRNA-133* genes are direct transcriptional targets of muscle differentiation regulators including serum response factor, MyoD or Mef2, suggesting a common set of regulatory elements that control cardiac and skeletal muscle development [41,42].

Dicer, an endoribonuclease of the RNase III family, is involved in the maturation of most miRNAs, and therefore might have significant impact on several biological processes. Dicer has been shown to be required for normal skeletal muscle development [40]. Interestingly, cardiac-specific deletion of Dicer results in defective heart development and embryonic lethality [31]. Similarly, Drosha/Dgcr8 containing complex also plays a role in miRNA processing. Cardiomyocyte-specific deletion of *dgcr8*, a gene required for microRNA biogenesis, revealed left ventricular malfunction progressing to a dilated cardiomyopathy and premature lethality, therefore, further highlighting the importance of miRNAs in heart development and function [32].

miRNA-133a negatively regulates cardiomyocyte proliferation during heart development. Over-expression of miRNA-133a driven by the βMHC promoter in embryonic cardiomyocytes inhibits cardiomyocyte proliferation and causes embryonic lethality characterized by a thinner ventricular wall [43]. On the contrary, miRNA-133a mutant mice exhibit excessive cardiomyocyte proliferation, attributed, in part, due to elevated expression of SRF and cyclin D2, which are targets for repression by miRNA-133a [43]. In another interesting study, hearts of miRNA-17–92-deficient mouse embryos presented a clear ventricular septal defect and severely hypoplastic lungs, suggesting critical roles for the miRNA-17–92 cluster during heart development [44]. Recent analysis identified miRNAs expressed in undifferentiated mouse embryonic stem cells and differentiating cardiomyocytes and found increased level of miRNA-1, miRNA-18, miRNA-20, miRNA-23b, miRNA-24, miRNA-26a, miRNA-30c, miRNA-133, miRNA-143, miRNA-182, miRNA-183, miRNA-200a/b, miRNA-292-3p, miRNA-293, miRNA-295 and miRNA-335 in mice [14,45]. Thus, miRNAs appear to play important roles in orchestrating organogenesis and early embryonic patterning processes.

Understanding the mechanisms of heart development may provide novel information for cardiac reprogramming technology therefore promoting newer therapeutic approaches for heart disease in the future.

### 3.2. miRNAs in Cardiac Hypertrophy

Cardiac hypertrophy is defined as enlargement, or thickening of the interventricular wall and/or septum, resulting from increases in cardiomyocyte size and changes in heart muscle signaling components [46,47,48,49,50,51,52]. Physiological hypertrophy is growth of heart, in response to exercise training or physiological needs like postnatal development and pregnancy, and an adaptive response that leads to stimulation of cardioprotective signaling cascades. In contrast, heart enlargement in response to cardiovascular diseases is broadly referred to as pathological hypertrophy and often progresses to heart failure [53].

To demonstrate the role of miRNAs in physiological hypertrophy in humans, Mooren *et al.* (2014) [54] investigated heart/muscle specific and inflammation related miRNAs in plasma of individuals before, directly after, and 24 h after a marathon run and correlated their relation to conventional biochemical, cardiovascular, and performance indexes. Interestingly, muscle specific miRNA, miRNA-1 showed a moderate negative correlation with fractional shortening, whereas miR-133a was positively related to the thickness of the intraventricular septal wall [54].

Intriguingly, analysis of the global expression of microRNAs in an experimental model of physiological left ventricular hypertrophy in mice showed an increase in miR-150 levels after 35 days and a decrease in miRNA-26b, miRNA-27a and miRNA-143 after 7 days of voluntary exercise [55]. Furthermore, the authors show that previously established regulatory gene pathways involved in pathological left ventricular hypertrophy are not changed in physiological left ventricular hypertrophy [55]. These studies suggest that understanding the role of miRNA in physiological hypertrophy might provide novel information that could be used to reverse the pathological hypertrophy process.

Studies using arrays to determine global expression of miRNAs have shown that pathological cardiac hypertrophy modulates miRNA expression [45,56,57,58,59,60,61,62]. These studies indicate that miRNAs, miRNA-208, miRNA-23a, miRNA-24, miRNA-125, miRNA-21, miRNA-129, miRNA-195, miRNA-199, and miRNA-212 are frequently increased in response to cardiac hypertrophy, whereas, miRNA-29, miRNA-1, miRNA-30, miRNA-133, and miRNA-150 expression are often found to be decreased. Therefore, forced expression of individual miRNAs for example miRNA-195 was sufficient to exacerbate pathological cardiac hypertrophy when over expressed in transgenic mice [16]. miR-195 targets mouse protein-25 (MO25), a central component of the MO25/Ste20 Related Adaptor (STRAD)/liver kinase B1 (LKB1) complex that acts as an upstream kinase for adenosine monophosphate-activated kinase (AMPK), a prominent player in the development of cardiac hypertrophy and heart failure [63]. In another study, *in vitro* over-expression of miRNA-150 and miRNA-181b, which are down-regulated in cardiac hypertrophy, resulted in reduced cardiomyocyte cell size [57]. Also, transgenic over-expression of miR-208a in the heart was sufficient to induce hypertrophic growth in mice, which resulted in pronounced repression of the miR-208 regulatory target thyroid hormone-associated protein 1 and myostatin, two negative regulators of muscle growth and hypertrophy [56]. Interestingly, suppression of miRNA-208a in heart failure rats prevents the pathological myosin switching while improving cardiac function [64]. Recent study has shown that pharmacological inhibition of miR-21 in a mouse model of Ang II–induced cardiac hypertrophy attenuated pathology [65]. These findings demonstrate an important role for specific miRNAs in the control of hypertrophic growth and remodeling of the heart in response to pathological signaling; therefore indicating that miRNAs might be targeted for therapy against heart disease.

### 3.3. miRNAs in Cardiac Fibrosis

Cardiac fibrosis is characterized by inappropriate deposition of extracellular matrix proteins in the myocardium leading to increased ventricular stiffness and contractile dysfunction, important contributors for the progression of heart failure in various pathophysiological conditions [46,47,48,49,51,52,66,67,68,69,70,71,72,73,74]. In addition, fibrosis causes disruption in electrical conductivity between cardiac myocytes, and therefore increasing susceptibility to arrhythmias [68,69,75].

Studies have shown that miRNA-21 is one of the most strongly up-regulated miRNAs in response to various forms of stress [16,57,76]. To demonstrate the role of miRNAs in cardiac fibrosis, Thum *et al.* (2008) has shown that miRNA-21 is up-regulated in cardiac fibroblasts in the failing heart and it represses the expression of Sprouty homologue 1 (Spry1) [77]. Furthermore, increased miRNA-21 augments extracellular signal-regulated kinase/mitogen-activated protein kinase signaling [77] or increased MMP-2 expression via decreased phosphatase and tensin homologue (PTEN) [78], leading to fibroblast proliferation and fibrosis. Intriguingly, cardiac fibroblasts have been shown to secrete miRNA-21-enriched exosomes that mediates cardiomyocyte hypertrophy through a paracrine signaling mechanism [65].

In our recent study, we demonstrated that miRNA-155 regulates cardiac fibrosis in diabetic (*db*/*db*) mice, possibly via inhibition of anti-fibrotic Sloan–Kettering Institute proto-oncogene (*Ski*) and Ski-related novel gene, non-Alu-containing (SnoN) signaling (negative regulators of TGF-β signaling) [6]. In another study, Duisters *et al.* (2009) [79] has demonstrated that miRNA-133 and miRNA-30, both consistently down regulated in several models of pathological hypertrophy and heart failure, regulate connective tissue growth factor (CTGF), a key molecule involved in fibrosis. Furthermore, *in vitro* experiments designed to modulate these miRNAs has been shown to effectively regulate CTGF expression by interacting directly with the 3' UTR region of CTGF mRNA [79].

Also, it has been shown that miR-29 family is down-regulated in the region of the heart adjacent to the infarct. The miR-29 family targets mRNAs that encode extracellular matrix-related proteins like collagens, fibrillins, and elastin, proteins involved in fibrosis [16]. Furthermore, down-regulation of miR-29 with anti-miRs *in vitro* and *in vivo* induced the expression of collagens [16]. These investigations indicate that miRNAs are important modulators of cardiac fibrosis and are involved in structural alteration during the progression of heart disease.

### 3.4. miRNAs in Cardiac Arrhythmias

Ischemic heart disease is a form of congestive heart failure that is caused by insufficient blood supply to the heart, resulting in loss of viable tissue. In response to injury, the non-ischemic myocardium displays signs of secondary remodeling, like interstitial fibrosis and hypertrophy. This remodeling process further deteriorates pump function and increases susceptibility to cardiac arrhythmias, in which the electrical activity of the heart is irregular resulting in significant morbidity and mortality. Muscle-specific miRNAs, miRNA-1 and miRNA-133 in addition to their role in cardiac development have been shown to be significantly up-regulated in ischaemic injury in the heart in both rodents and humans [75,80,81]. Over-expression of miRNA-1, in normal or infarcted rat hearts, slowed conduction and depolarized the cytoplasmic membrane by post-transcriptionally repressing *KCNJ2* (which encodes the K^+^ channel subunit Kir2.1) and *GJA1* (which encodes connexin 43), and therefore exacerbates arrhythmogenesis [75]. Recent studies indicate that connexin 43, a major cardiac gap junction protein, is a direct target of miRNA-130a and that over-expression of miRNA-130a may contribute importantly to gap junction remodeling and to the pathogenesis of atrial and ventricular arrhythmias [82].

Clinical and preclinical studies have shown that miRNA-212 is up-regulated during heart failure [45,83,84]. miRNA-212 targets Kir2.1 (KCNJ2 mRNA) that carries an inward rectifier K^+^ current critical for maintaining membrane potential. Studies have indicated that Pitx2 (homeobox transcription factor) insufficiency leads to atrial electrical and structural remodeling linked to arrhythmogenesis [85,86]. Intriguingly, Pitx2 has been shown to positively regulate miRNA-17–92 and miRNA-106b-25. Furthermore, intracardiac electrical stimulation revealed that both miRNA-17–92 and miRNA-106b-25 deficient mice exhibit pacing-induced atrial fibrillation, the most common sustained cardiac arrhythmia [87].

A recent study investigated the relation of reduced expression of miRNA-150 in platelets to atrial fibrillation in patients with chronic systolic heart failure. The authors concluded that miRNA-150 expression levels in platelets of patients with systolic heart failure with atrial fibrillation are significantly reduced and correlated to the cell-free circulating levels of this miRNA. However, the role of miRNA-150 in atrial fibrillation has not been completely understood [88]. Interestingly, in a rabbit model of diabetes, miRNA-133 was shown to be up-regulated in the heart in association with increased expression of serum response factor, which is known to be a transactivator of miRNA-133 [89]. The authors further show that miRNA-133 represses ERG (*ether-a-go-go-related gene*) leading to depressed *I*_Kr_, slow repolarization and QT prolongation associated with arrhythmias in diabetic hearts.

Diabetic patients are at increased risk for heart failure [90]. A recent study has shown that hyperglycemia augmented expression of miRNA-1 and miRNA-133 in human cardiac progenitor cells associated with suppressed KCNE1 and KCNQ1 and significant reduction in the functional *I*_Ks_ current [91]. Furthermore, addition of miRNA-1 and miRNA-133 antagomirs diminished the inhibitory effect of high glucose on KCNE1 and KCNQ1 and restored the potassium current *I*_Ks_ [92].

### 3.5. miRNAs in Endothelial Homeostasis and Angiogenesis

Endothelial cell integrity and function is fundamental for homoeostasis of the vascular system. The induction of new blood vessel formation is an orchestrated process, which is critical during development and tissue repair in response to injury. A recent study suggests that miRNA-26a regulates pathological and physiological angiogenesis by targeting endothelial cell (EC) bone morphogenic protein/SMAD1 signaling *in vitro* and *in vivo* [93]. Interestingly, systemic intravenous administration of a miRNA-26a inhibitor increased SMAD1 expression and rapidly induced angiogenesis associated with improved heart function [93]. Local delivery of adenovirus-mediated miRNA-24 decoy in the ischemic area of myocardium increased angiogenesis and blood perfusion [94]. Human endothelial cells from advanced neovascularized atherosclerotic lesions showed that miRNA-222 expression was negatively correlated to signal transducer and activator of transcription 5A (STAT5A) expression and diminished proliferation and vessel formation ability [95]. Knockdown of miRNA-221 and miRNA-222 suppressed vascular smooth muscle cell (VSMC) proliferation and neointimal lesion formation after carotid angioplasty [96]. miR-210 over-expression stimulates tubulogenesis and migration by targeting receptor tyrosine-kinase ligand Ephrin-A3 [92]. Chen and Gorski [97] showed that miRNA-130a regulates angiogenic phenotype of vascular ECs through down-regulation of anti-angiogenic homeobox genes GAX and HOXA5 [97]. Intriguingly, endothelial cell specific miRNA, miRNA-126-mediated phosphoinositide-3-kinase regulation stimulates VEGF-signaling and strongly enhances the activities of Ang-1 on vessel stabilization and maturation [98]. These studies suggest that regulating miRNAs involved in endothelial cell biology and function serves as potential therapeutic target to treat diseases with vascular dysfunction.

## 4. miRNAs in Cardiac Regeneration and Stem Cell-Mediated Repair

Coronary artery occlusion with cycles of ischemia and reperfusion leads to chronic inflammation and oxidative stress resulting in significant loss of cardiac myocytes and endothelial cells, causing impairment in cardiac function and heart failure. The existing therapies slow down the pathogenesis of cardiovascular diseases, however, the long-term survival of patients with heart failure depends on regenerating the lost cardiac tissue and reestablishing perfusion in the myocardium, therefore leading to improved repair of the heart tissue. In this direction, recent concepts for myocardial regeneration include: stimulating terminally differentiated cardiomyocyte to proliferate, stem/progenitor cell transplantation, direct reprogramming of scar tissue into functional myocardium and tissue engineering.

Shortly after birth, cardiomyocytes stop proliferating, therefore, their ability to regenerate in response to injury is very limited [99]. Intriguingly, recent evidences indicate that cardiomyocytes are able to proliferate during the postnatal life [100,101]. Porrello *et al.* [102] demonstrated that the miRNA-15 family of miRNAs modulates neonatal heart regeneration through inhibition of postnatal cardiomyocyte proliferation. Further analysis showed that miRNA-195 (a member of miRNA-15 family) regulates expression of a number of cell cycle genes, including checkpoint kinase 1 (Chek1), which was identified as a highly conserved direct target of miRNA-195 [102]. Most recently, using both transgenic and knockout mice models, Chen *et al.* (2013) [103] demonstrated that miRNA-17-92 cluster (specifically, through miRNA-19a/b-mediated inhibition of the tumor suppressor gene, PTEN) is required for and sufficient to induce cardiomyocyte proliferation in postnatal and adult hearts. These studies suggest that the miRNAs identified hold great promise for the treatment of cardiac pathologies consequent to cardiomyocyte loss.

A recent strategy to directly reprogram fibroblasts into cardiomyocytes using a combination of three or four cardiac-specific transcriptional factors, Gata4, Mef2c, Tbx5 and/or Hand2, *in vitro* [104] and *in vivo* [105,106] is an exciting breakthrough. Reports indicate that miRNAs in synergy with cardiac transcriptional factors might regulate reprogramming process. Jayawardena *et al.* (2012) [107] demonstrated that miRNA-1 is sufficient to induce reprogramming of fibroblast into cardiomyocytes, however, the efficiency was significantly enhanced by adding miRNAs-133, -208 and -499 and JAK inhibitor I. Importantly, administration of these miRNAs into ischemic mouse myocardium resulted in evidence of direct conversion of cardiac fibroblasts to cardiomyocytes *in situ* [107].

Following myocardial infarction, successful formation of new vasculature in the injured myocardium is important to restore myocardial perfusion and therefore cardiac function. Meloni *et al.* (2013) [94] have shown that myocardial infarction induction in mice decreased miRNA-24 expression in the peri-infarct tissue and its resident cardiomyocytes and fibroblasts; while it is increased in endothelial cells (ECs). *In vitro*, miRNA-24 inhibition enhanced human ECs survival, proliferation and networking in capillary-like tubes in association with increased eNOS (a direct target of miRNA-24) [94]. In another study, endothelial cell specific miRNA-126 has been shown to impair ischemia-induced angiogenesis in a hind limb ischaemia model [108]. miRNA-126 functions in part by directly repressing negative regulators of the VEGF pathway, including the Sprouty-related protein SPRED1 and phosphoinositol-3 kinase regulatory subunit 2 (PIK3R2/p85-β) [109].

Recent studies have shown that miRNAs are dysregulated in early EPCs derived from patients with cardiovascular disease, which may critically limit the endogenous repair response. Early EPCs derived from patients with coronary artery disease have shown an increase in miRNAs like miRNA-21 [110], and miRNA-221/222 [111]. Moreover, over-expression of miRNA-21 in early EPCs has been shown to inhibit their migratory capacity through repression of superoxide dismutase 2 (a key protection protein against oxidative damage) and inhibition of endogenous mitogen-activated protein kinase inhibitor, sprouty-2 [110]. In another study, over-expression of miRNA-34a (mimic) inhibited EPC-mediated angiogenesis by inducing senescence via suppressing silent information regulator 1 (Sirt1) [112].

## 5. Challenges of MicroRNA-Based Therapies in Cardiovascular Diseases

Although targeting miRNAs has been used as novel therapeutic strategy, miRNA-based therapeutics faces multiple challenges. Since one miRNA might regulate a large number of targets and each gene can also be regulated by several different miRNAs, targeting of a miRNAs might potentially perturb multiple cellular functions [113]. Furthermore, this might lead to off-target effects of unidentified miRNA targets. As with any drug, the *in vivo* delivery of miRNA modulators and organ or tissue specific targeting is a challenge and therefore might require an addition of modulators such as cell surface receptor ligands and nucleic acids that can enhance target binding to the tissue or cell type of interest [114]. In addition, short half-life and/or chemical modification of these agents might alter the biological properties and might also pose toxicity issues [114]. Despite these challenges, the use of miRNAs hold great promise, since these molecules are naturally occurring endogenous regulators of cell processes that are often dysregulated in disease. These challenges could be overcome by further in-depth understanding of the miRNA biology and regulation in different disease processes and validation of target mRNAs. Also, development of more selective and stable mimics and modulators of miRNA, could lead to better approaches to target miRNAs in various diseases.

## 6. Clinical Perspectives and Conclusions

**Figure 1:**
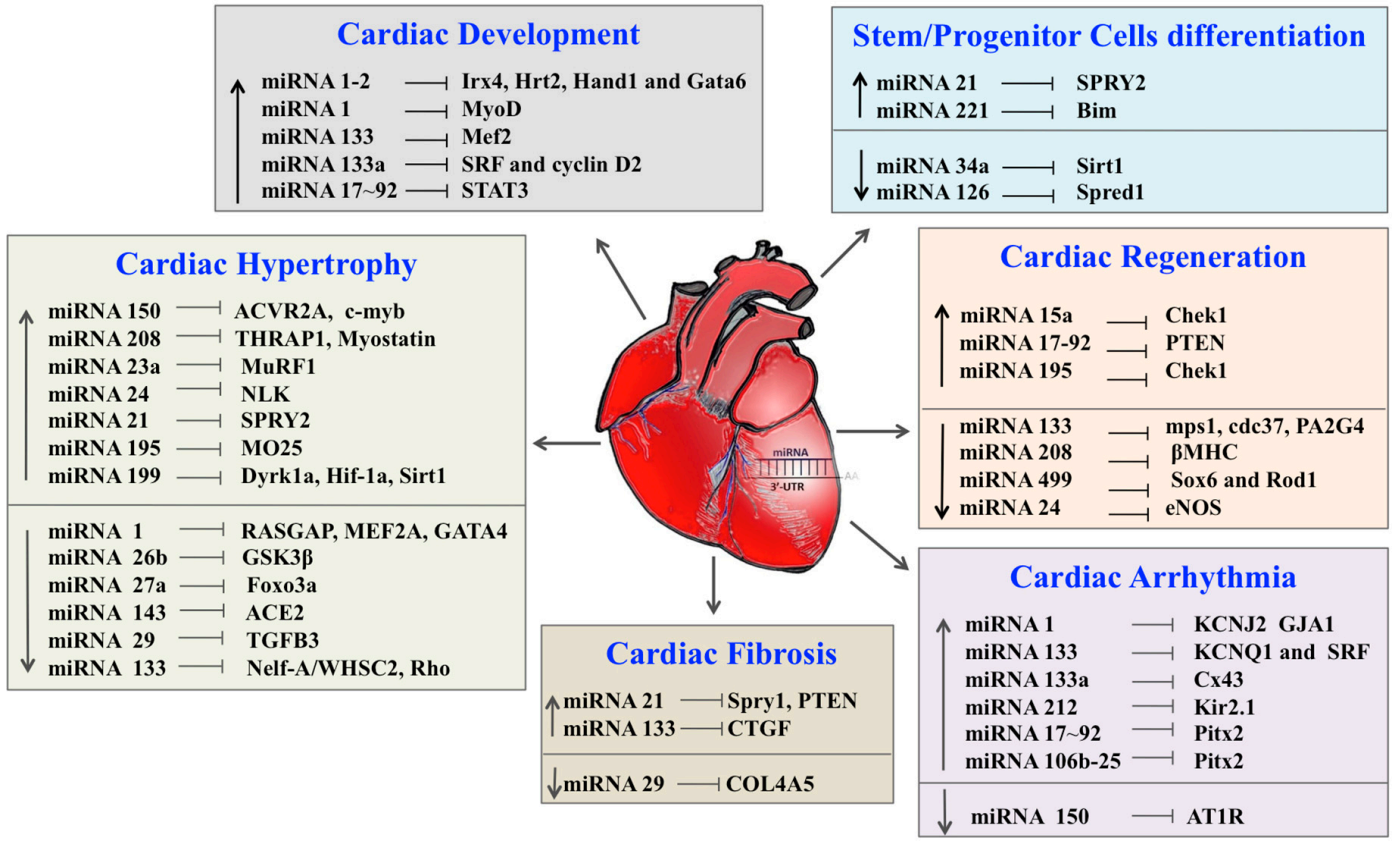
Role of miRNAs in cardiac development, disease and regeneration. **↑** Increasing, **↓** Decreasing, ⟞ Inhibiting.

Numerous studies have shown that cardiac and circulating miRNAs are markedly altered in patients with heart failure [115,116,117]. miRNAs have emerged as a novel class of key regulators in various biological processes involved in cardiovascular diseases like fibrosis, hypertrophy, endothelial homeostasis, arrhythmias, stem cell-mediated repair and cardiac regeneration (summarized in Table 1 and Figure 1). As a result of tissue hypoxia, several key processes, such as inflammation; angiogenesis; cell death by apoptosis, autophagy and necrosis; fibrosis and hypertrophy are activated. miRNAs have been implicated in all of the above processes and might be released into the circulation, and therefore, serve as novel and sensitive biomarkers of cardiac damage [118]. Interestingly, these miRNAs might also be packaged within exosomes and microvesicles and circulate in a stable form in many body fluids, including blood, suggesting that they might possibly act as key molecules in cell-to-cell communications and signaling [28]. Interestingly, miRNA expression in EPCs was associated with the prognosis of chronic heart failure secondary to ischemic cardiomyopathy or non-ischemic cardiomyopathy [119]. These studies suggest that detection of circulating miRNAs could be used as diagnostic and/or prognostic markers of cardiovascular diseases. Also, considering the potential role of miRNAs in a multitude of human diseases, miRNAs and their respective targets may represent an exciting prospect for therapeutic applications to limit cardiac damage and promote tissue regeneration after myocardial infarction, stroke, or other ischemic events.

Therefore, understanding miRNA biogenesis, their interactions with regulatory signaling cascades and miRNA profiling in cardiac diseases is not only necessary for an optimized, targeted therapy, but might also provide novel opportunities to dissect their key role in cardiovascular development, homeostasis, disease pathogenesis, relevant stem cell function and regeneration.

**Table 1:** Potential targets of miRNA associated with cardiovascular biology.

miRNA	Targets	References
**Cardiac Development**		
miRNA-1–2	Irx4, Hrt2, Hand1 and Gata6	[120]
miRNA-1	MyoD, Hand2	[41,42]
miRNA-133	Mef2	[41,42]
miRNA-133a	SRF and cyclin D2	[43]
miRNA-17–92	STAT3	[44,121]
miRNA-20	Egln3	[122]
miRNA-23b	Rb phosphorylation	[123]
miRNA-24	BIM and GATA	[94]
miRNA-30c	CTGF	[79]
miRNA-143	Adducin3	[124]
**Cardiac Hypertrophy**		
miRNA-150	ACVR2A, c-myb	[125]
miRNA-208	THRAP1, Myostatin	[56]
miRNA-23a	MuRF1	[126]
miRNA-24	NLK	[127]
miRNA-21	SPRY2	[128]
miRNA-195	MO25	[63]
miRNA-199	Dyrk1a, Hif-1a, Sirt1	[129,130,131,132]
miRNA-1	RASGAP, MEF2A, GATA4	[60,133]
miRNA-26b	GSK3β	[134]
miRNA-27a	TGF-β1	[135]
miRNA-143	ACE2	[136]
miRNA-29	TGFB3	[127]
miRNA-133	Nelf-A/WHSC2, Rho	[137]
**Cardiac Fibrosis**		
miRNA-21	Spry1, PTEN	[77]
miRNA-133	CTGF	[79]
miRNA-29	COL4A5	[16]
**Cardiac Arrhythmia**		
miRNA-1	KCNJ2 GJA1	[75]
miRNA-133	KCNQ1 and SRF	[138]
miRNA-133a	Cx43	[82]
miRNA-212	Kir2.1	[83,84]
miRNA-17–92	Pitx2	[87]
miRNA-106b	Pitx2	[87]
miRNA-150	AT1R	[88]
**Cardiac Regeneration**		
miRNA-15a	Chek1	[102]
miRNA-17–92	PTEN	[103]
miRNA-195	Chek1	[102]
miRNA-133	mps1, cdc37, PA2G4, cx43, cldn5	[139]
miRNA-208	βMHC	[41]
miRNA-499	Sox6 and Rod1	[115]
miRNA-24	eNOS	[94]
**Stem/Progenitor Cells Differentiation**		
miRNA-21	SPRY2	[110]
miRNA-221	Bim	[140]
miRNA-34a	Sirt1	[112]
miRNA-126	Spred1	[109,141]
